# Photoelectrochemical, impedance and optical data for self Sn-diffusion doped Fe_2_O_3_ photoanodes fabricated at high temperature by one and two-step annealing methods

**DOI:** 10.1016/j.dib.2015.10.031

**Published:** 2015-11-06

**Authors:** Pravin S. Shinde, Alagappan Annamalai, Ju Hun Kim, Sun Hee Choi, Jae Sung Lee, Jum Suk Jang

**Affiliations:** a Division of Biotechnology, Advanced Institute of Environmental and Bioscience, College of Environmental and Bioresource Sciences, Chonbuk National University, Iksan 570-752, Republic of Korea; b School of Energy and Chemical Engineering, Ulsan National Institute of Science and Technology (UNIST), 50 UNIST-gil, Ulsan 689-798, Republic of Korea; c Pohang Accelerator Laboratory (PAL), Pohang University of Science and Technology (POSTECH), Pohang 790-784, Republic of Korea

**Keywords:** Hematite thin films, PRED, Sn diffusion doping, Photoelectrochemical, Water oxidation

## Abstract

The optical, morphological and photoelectrochemical (PEC) properties of transition metal oxide semiconductors are important to understand their influence on water oxidation performance. Herein, we provide experimental evidences for a better understanding of the factors that dictate the interactions of Sn-diffusion doping on the PEC properties of Fe_2_O_3_ photoanodes fabricated at high temperature by one- and two-step annealing methods. The synthesis, characterization methods and other experimental details are provided. Limited previous information on the PEC and electrochemical impedance spectroscopic studies has been published. This data article contains Supplementary data, figures and methods related to the research article by Shinde et al. (2015) [1]. Here, we provide a further set of the obtained experimental data results.

**Specifications Table**

**Table:** 

Subject area	*Physics, Chemistry*
More specific subject area	*Photoelectrochemical water splitting*
Type of data	*Image, graph*
How data was acquired	*Potentiostat (COMPACTSTAT.e, Ivium, Netherland) equipped with impedance analyzer, solar simulator (Abet Technologies), field emission scanning electron microscope (SUPRA 40VP, Carl Zeiss, Germany), thermo scientific XPS spectrometer, dual beam UV–vis–NIR spectrophotometer (Shimadzu, UV-2600 series)*
Data format	*Analyzed*
Experimental factors	*J–V measurements of α-Fe*_*2*_*O*_*3*_*/FTO photoanodes fabricated at 800 °C by one- and two-step annealing methods. Co-relation of electrochemical impedance and optical absorption behavior of photoanodes with the water splitting performance.*
Experimental features	*Optimization of one-step annealing temperature and annealing time based on photocurrent density at 1.23V* vs *RHE.*
Data source location	*Division of biotechnology, advanced institute of environmental and bioscience, college of environmental and bioresource sciences, Chonbuk National University, Iksan, Republic of Korea*
Data accessibility	*Data are available within this article and are related to*[1]

**Value of the data**

• Activation of α-Fe_2_O_3_/FTO photoanode for prominent photoelectrochemical water oxidation performance can effectively be accomplished by means of Sn-diffusion doping via underlying FTO substrate at 800 °C.
• The data provide the insightful *J*–*V* characteristics comparison of α-Fe_2_O_3_/FTO photoanodes fabricated by a rapid one-step annealing over a conventional tedious two-step annealing process, highlighting the importance of annealing methodology.
• The *J*–*V* characteristics data provide information about early onset potential and higher photocurrent response measured at 1.23 V_RHE_ for α-Fe_2_O_3_/FTO photoanodes on account of one-step annealing.
• Nyquist plots data for all photoanodes under study can be used to account for interfacial charge transfer processes and FTO deformation resulting from different annealing conditions, which can be co-related with the water oxidation photocurrent behavior of photoanodes.

## 1 Data, experimental design, materials and methods

In the following sections, we have presented a detailed description about how to synthesize efficient α-Fe_2_O_3_ photoanodes with their potential application in photoelectrochemical water oxidation for hydrogen generation. Optimization of photoanodes fabricated at high temperature (800 °C) by one- and two-step annealing methods is carried out based on their water oxidation photocurrent performance at water splitting potential of 1.23 V vs RHE. Finally, the optical absorption data are co-related to account for the photocurrent response of fabricated photoanodes.

### 1.1 Cleaning of FTO substrate

Substrate cleaning is an important step in electrodeposition. Transparent conducting glass (fluorine-doped tin oxide, FTO, 10–15 Ω cm^−1^) substrates were used to prepare iron (oxide) thin films. FTO substrates were cut into a required dimension (1 cm×2.5 cm) suitable for electrodeposition. The cut substrates were successively cleaned in acetone, ethanol and deionized water in ultrasonic bath each for 10 min. Finally, the cleaned substrates were dried using nitrogen (N_2_) stream until all the water drops or any dusts are removed. The drying with N_2_ was performed just before the FTO substrates are used for electrodeposition of iron (oxide) films.

### 1.2 Materials and methods

The iron(oxide) photoanodes were prepared by a facile pulse reverse electrodeposition (PRED) method as reported previously [2]. In brief, Fe thin films (~200 nm) were grown on as-cleaned FTO by PRED in a two-electrode cell configuration involving FTO as a working electrode and convoluted platinum wire as counter electrode. The electrolyte consisted of 6 g ferrous sulfate, 0.15 g ascorbic acid, 0.05 g amidosulfonic acid and 1.5 g boric acid in 0.1 L deionized water (pH=5.71). The pH of the resulting electrolyte solution was 2.6. The chemicals were purchased from Alfa Aesar and Kanto chemicals, and used without further treatment. The applied potential and pulse parameters were controlled using a function generator (Agilent 33220A, 50 MHz). The process parameters of PRED such as amplitude of square wave pulse, duty cycle, pulse period, and deposition time were [10 V (−6/+4 V)], 20%, 10 ms, and 45 s, respectively. The as-grown Fe films were rinsed several times in deionized water and dried immediately using N_2_ stream. As-grown films were dark greyish in color and reflected the light. At least three electrodes of each condition were prepared.

### 1.3 Fabrication of α-Fe_2_O_3_ photoanodes

**Fig. 1:**
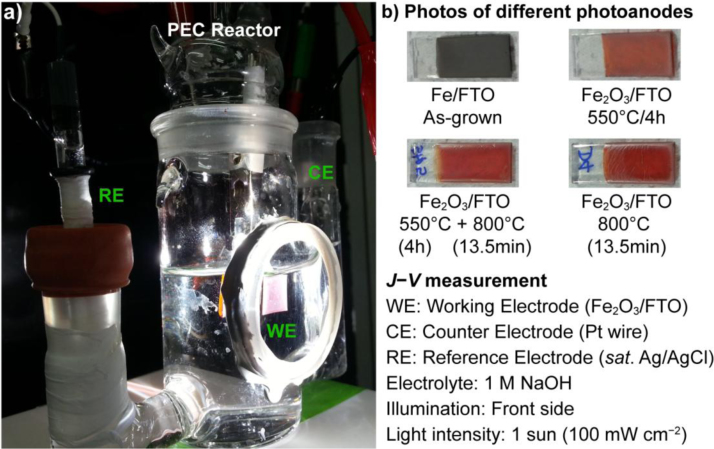
(a) PEC reactor used for *J*–*V* and impedance measurements and (b) Photo-images of as-grown Fe and annealed Fe_2_O_3_ films synthesized by a PRED method.

The conversion of Fe films to photoactive Fe_2_O_3_ on FTO was accomplished by annealing at high temperature (HT). A direct one-step HT-annealing method was employed. It involved inserting the as-grown samples into the box furnace (whose temperature was already attained to HT, viz. 700≥*T*≤825 °C), soaked for a short duration, air-quenched for few seconds and then transferred to a forced-convection oven at 100 °C for 10 min. After 10 min, the samples were taken out to ambient air for cooling down completely. By following such a mild air-quenching process, we avoided the breaking of high-temperature-annealed red-hot samples as well as maintained the crystalline synthesis of hematite photoanodes. For comparison, Fe_2_O_3_ films were also fabricated by two-step annealing approach. In this method, during first step, the as-grown films were heated to 550 °C at the rate of 5 °C min^−1^ and baked there for 4 h, and cooled naturally until room temperature. In the second step, the films were annealed at 800 °C briefly for 15 min, quenched in air and subsequently transferred to an oven at 100 °C for 10 min. The annealed films were reddish or dark brown in color. The Fe_2_O_3_ films fabricated by two-step annealing are smoother than those prepared by one-step annealing method. The later films clearly show the wrinkled surface as seen from Fig. 1. Despite of having little wrinkled surface, one-step-annealed films were well-adherent and showed no signs of any degradation. The Fe_2_O_3_ photoanodes fabricated by one-step annealing method were investigated further due to their advantage over two-step annealing method in terms of cost-effective synthesis. For one-step annealing, several as-grown films under identical synthesis conditions were prepared by PRED to investigate the effects of annealing temperature and annealing time. Initially, by keeping the annealing time fixed for 15 min, the elevated temperature for one-step annealing was varied from 700 to 825 °C. In the next step, by choosing the optimized annealing temperature, the annealing time was varied from 10 to 17.5 min to decide on the optimum growth time for improving the photocatalytic activity of iron oxide. The annealing conditions of samples (iron oxide thin films) in both cases were optimized by means of their PEC performance measured at 1.23 V vs RHE.

## 2 Characterization of α-Fe_2_O_3_ photoanodes

The α-Fe_2_O_3_ photoanodes fabricated by one- and two-step annealing methods were characterized by *J*–*V* characteristics, surface morphology, electrochemical impedance spectroscopy, X-ray photoelectron spectroscopy and UV–vis spectroscopy.

### 2.1 *J–V* characteristics

A typical PEC reactor used for measurement of *J*–*V* curves of fabricated photoanodes is shown in Fig. 1. The *J*–*V* measurements of α-Fe_2_O_3_/FTO photoanodes fabricated at 800 °C by one- and two-step annealing methods were carried out in a PEC reactor consisting of three-arm glass compartment with a circular quartz window for light illumination. A simulated 1 sun (100 mW cm^−2^) light illumination was provided from front-side using a solar simulator (Abet Technologies). The PEC cell comprised of Fe_2_O_3_/FTO photoanode as working electrode, Pt wire as counterelectrode, Ag/AgCl (saturated with KCl) as reference electrode immersed in 1 M NaOH electrolyte. Only 1×1 cm^2^ area of photoanode under investigation was immersed and exposed to light by covering the rest with a Teflon tape. All the potentials mentioned in this work were measured with reference to Ag/AgCl electrode and were revised for the reversible hydrogen electrode (RHE) scale using the Nernst equation as below [3]:

(1) $$E_{\mathrm{RHE}}=E_{\mathrm{Ag}/\mathrm{AgCl}}+0.059\;\mathrm{pH}+E_{\mathrm{Ag}/\mathrm{AgCl}}^{o}$$

where $V_{\mathrm{RHE}}$ was the converted potential vs RHE, $V_{\mathrm{Ag}/\mathrm{AgCl}}^{o}$=0.1976 V at 25 °C, and $V_{\mathrm{Ag}/\mathrm{AgCl}}$ was the experimental potential against the Ag/AgCl electrode.

**Fig. 2:**
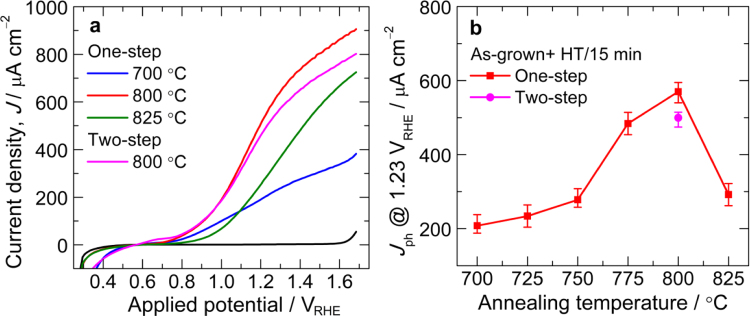
(a) Representative current density–voltage (*J*–*V*) curves of α-Fe_2_O_3_ photoanodes fabricated with different one-step annealing temperatures for 15 min and (b) Variation of photocurrent density (*J*_ph_) measured at 1.23 V_RHE_ for all the photoanodes. The error bars represent the standard deviations of the PEC measurements of the independently prepared series of hematite photoanodes. Light illumination, 1 sun; scan rate, 50 mV s^−1^. For comparison, *J*–*V* curve and *J*_ph_ value at 1.23 V_RHE_ of α-Fe_2_O_3_ photoanode prepared by two-step annealing methods with similar HT annealing condition (e.g. 800 °C for 15 min) are shown in a and b, respectively.

**Fig. 3:**
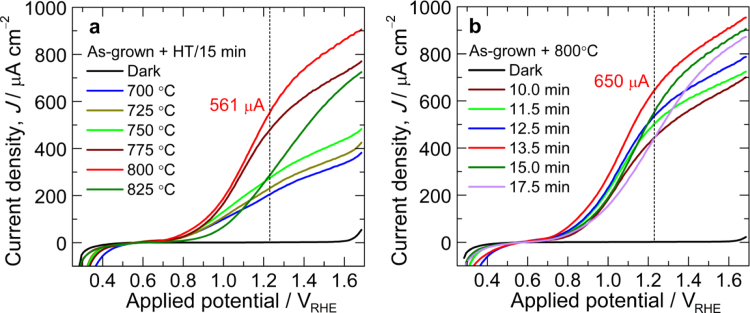
Current density–voltage (*J*–*V*) curves of α-Fe_2_O_3_ photoanodes fabricated from as-grown Fe/FTO by one-step-annealing (a) at different high temperatures for 15 min and (b) at fixed 800 °C for different annealing times.

**Fig. 4:**
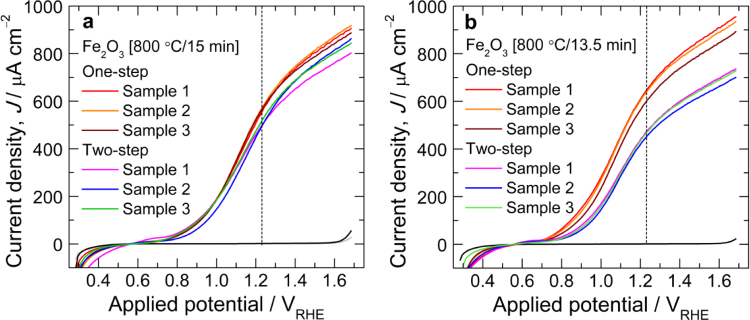
*J*–*V* curves of α-Fe_2_O_3_ photoanodes fabricated by one-step (800 °C) and two-step (550 °C/4 h +800 °C) annealing method, with HT-annealing at 800 °C for (a) 15 and (b) 13.5 min.

The photoactivity of hematite fabricated on FTO (F:SnO_2_) substrate at low temperature is very poor and requires a thermal activation at high temperatures (650–800 °C) to induce Sn doping from FTO for photocurrent improvement. Therefore, to decide the optimum annealing temperature, first we systematically varied the annealing temperature with a fixed annealing duration of 15 min. One-step annealing temperature of 800 °C for 15 min is found to be optimum temperature based on its higher photocurrent output at 1.23 V vs RHE among the studied annealing temperatures. This photocurrent is even higher than that of two-step annealed α-Fe_2_O_3_ photoanode fabricated with similar high-temperature annealing condition (800 °C for 15 min) as seen from Fig. 2a and b. The photocurrent density of α-Fe_2_O_3_ photoanode fabricated by one-step annealing at 700, 725, 750 and 825 °C was much lower than those of 775 and 800 °C. The difference is clearly visible at higher applied potentials (Fig. 3a). After optimizing the annealing temperature, the annealing duration was varied as shown in Fig. 3b. It is clear that annealing duration of 13.5 min is optimum to deliver optimum photocurrent (650 μA cm^−2^) from α-Fe_2_O_3_ photoanode fabricated at 800 °C. Fig. 4a and b shows the *J*–*V* curves of α-Fe_2_O_3_ photoanodes fabricated by one-step (800 °C) and two-step (550 °C/4 h+800 °C) annealing methods, with high-temperature annealing at 800 °C for 15 and 13.5 min, respectively. Data for at least three samples fabricated under identical condition are measured for each parameter. As seen from the figures, the close match of the curves affirms the reproducibility of the data.

### 2.2 Surface morphological characterization

**Fig. 5:**
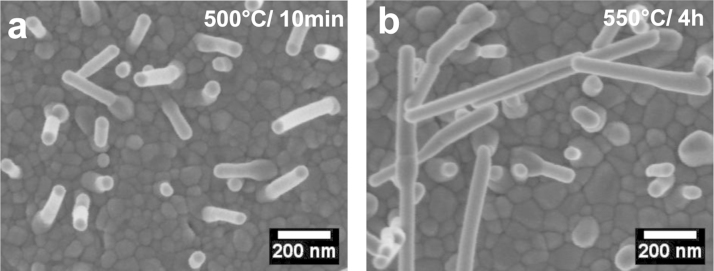
FESEM images of iron oxide films deposited at 45 s PRED time and annealed at 500 °C for (a) 10 min and (b) 4 h, revealing grain growth, inter-connectivity of grains and nano-structured morphology.

The surface morphology of the low-temperature annealed α-Fe_2_O_3_ films was examined on field emission scanning electron microscope (FESEM, SUPRA 40VP, Carl Zeiss, Germany). Fig. 5 shows the top view of α-Fe_2_O_3_ films grown on FTO fabricated by conventional annealing at 550 °C for 10 min and 4 h. As seen from figure, the morphology consists of compactly arranged nano-crystalline grains with good inter-connectivity. Apparently, the grain size of α-Fe_2_O_3_ annealed for 4 h is relatively higher than that annealed for short duration of 10 min. The 200 nm long and 50 nm thick nano-rods that originate from the surface also appear to grow further (up to ~700 nm). The annealing at high temperature by one- or two-step methods collapses the nanostructures and brings about further grain growth [1].

### 2.3 XPS analysis

The chemical state and elemental quantification in the freshly synthesized iron oxide samples was performed using X-ray photoelectron spectroscopy (XPS). The XPS analysis was performed on a Thermo Scientific XPS spectrometer equipped with a monochromatic Al K*α* X-ray source (*hν*=1486.6 eV). Wide survey spectra (Binding Energy, BE: 1200–0 eV) were recorded for the samples using the X-ray spot size of 400 µm at room temperature with an analyzer pass energy of 200 eV and energy step size of 1 eV.

**Fig. 6:**
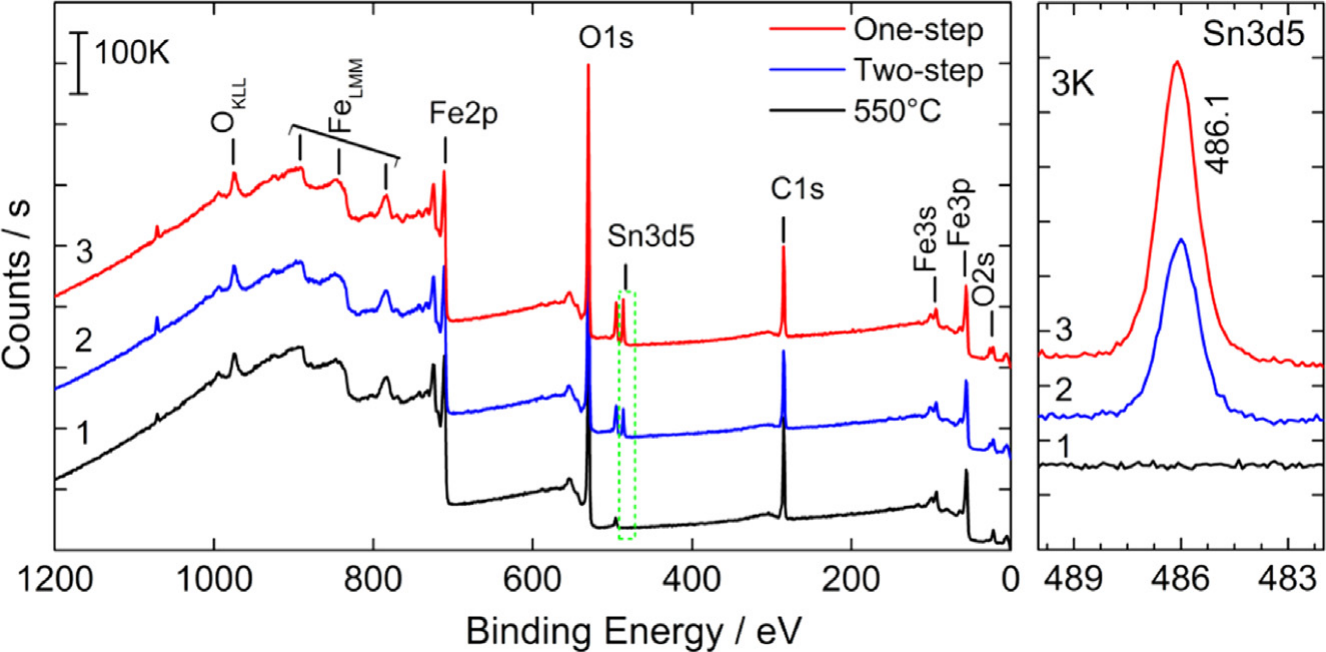
Survey XPS spectra of different iron(oxide) samples prepared on FTO substrate. Samples: (*curve* 1) low temperature annealing at 550 °C for 4 h, (*curve* 2) two-step annealing at 550 °C followed by 800 °C, and (*curve* 3) one-step annealing at 800 °C.

Fig. 6 shows the XPS survey spectra of iron oxide samples fabricated by low-temperature (550 °C/4 h), two-step (550 °C/4 h+800 °C/13.5 min) and one-step (800 °C/13.5 min) annealing methods. The binding energy (BE) peaks centered at 711, 530, 486, 285, 94, 56 and 23 eV are assigned to Fe 2p, O 1s, C 1s, Sn 3d, Fe 3s, Fe 3p, and O 2s photo-electrons, respectively. The broad peaks centered at 974, 894, 847 and 785 eV are assigned to the OKLL, FeLML and Auger transitions, respectively. Among all the samples, the Fe 2p_3/2_ peak is observed at a BE of 710.6 eV, and its absence at 709.3 eV suggests the oxidation state of Fe to be +3, confirming the formation of phase-pure α-Fe_2_O_3_. It is also apparent that the Sn 3d_5/2_ peak is absent for the 550 °C-annealed iron oxide sample, but shows its presence in two- and one-step-annealed samples. This is due to the high-temperature directed diffusion of Sn^4+^ ions from underlying FTO into the Fe_2_O_3_ matrix. The Sn enrichment can boost the electronic conductivity and hence improve the photoactivity of hematite [4]. The increased Sn 3d5 peak intensity for the one-step-annealed sample indicates a higher amount of diffused Sn content than that for the two-step-annealed sample.

### 2.4 EIS characterization

**Fig. 7:**
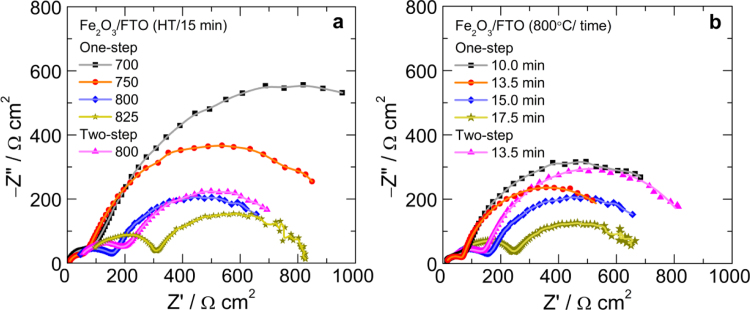
Nyquist plots of one-step-annealed hematite photoanodes fabricated with (a) different annealing temperatures and (b) annealing durations recorded in 1 M NaOH electrolyte under 1 sun illumination. For comparison, the Nyquist plots of two-step annealed photoanode fabricated with similar HT-annealing at 800 °C for 15 min and 13.5 min are also shown.

The charge transfer characteristics of hematite photoanodes were studied by electrochemical impedance spectroscopy (EIS) at 1.23 V_RHE_. Fig. 7a shows the Nyquist plots (real vs imaginary impedance) of one-step-annealed hematite photoanodes fabricated with different annealing temperatures for a fixed annealing duration of 15 min. The charge transfer resistance (expressed from diameter of Nyquist plot arc) of the photoanodes fabricated at 700 and 750 °C is higher, being lowest for 800 °C. The Nyquist plots of photoanodes fabricated at 800 and 825 °C are shifted towards higher impedance region, indicating the deformation of FTO substrate. More severe FTO deformation can be seen at 825 °C.

Fig. 7b shows the Nyquist plots of one-step-annealed hematite photoanodes fabricated at 800 °C with different annealing times. Annealing duration of 13.5 min is found be optimum as one can see lowest charge transfer resistance and less FTO deformation. For comparison, the Nyquist plots of two-step annealed photoanodes fabricated with similar HT-annealing at 800 °C for 15 min and 13.5 min are also shown. Two-step annealing with similar HT-annealing condition causes relatively more deformation resulting in higher charge transfer resistance. The EIS results are in concurrent with the *J*–*V* characteristics data of the respective photoanodes [1].

### 2.5 Optical characterization

**Fig. 8:**
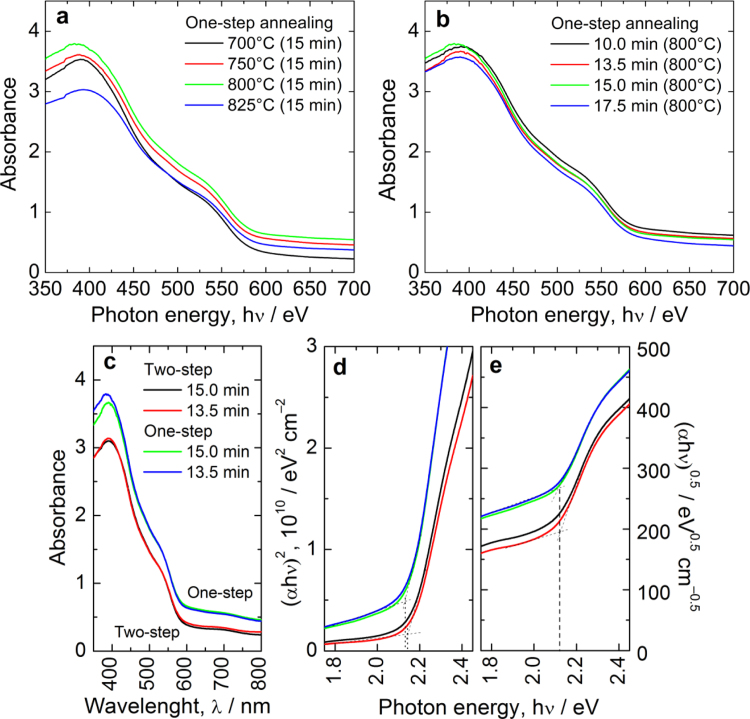
Plot of absorbance vs wavelength for one-step-annealed hematite photoanodes prepared with (a) different annealing temperatures for 15 min and (b) different annealing times at 800 °C. The plots of absorbance (c) and band gap energy (d and e) for one-step, and two-step-annealed hematite photoanodes. The figure legends in (c) are also applicable to (d and e). Tauc plots reveal the direct (*E*_g_~2.13–2.14 eV) and indirect (*E*_g_ ~2.11 eV) optical transitions.

The UV–vis absorption study in the wavelength range of 350–800 nm was performed using a dual beam spectrophotometer (Shimadzu, UV-2600 series). Fig. 8a and b shows the absorbance (measure of absorbed photons) of one-step-annealed hematite photoanodes at representative annealing temperatures and annealing times. Absorbance values at each wavelength are increased with HT-annealing from 700 to 800 °C but decreased for 825 °C (Fig. 8a). The reduced absorbance could be due to excess diffusion of Sn ions into the hematite lattice altering the optical properties. No significant change is noticed in the absorbance values for annealing time variation (Fig. 8b).

The absorbance of one-step-annealed photoanodes was higher than that of two-step-annealed hematite at each wavelength as shown in Fig. 8c and also exhibited significant response at wavelengths greater than the band gap (~590 nm) due to scattering caused by the large particles. Such a scattering effect was lower in the two-step-annealed hematite due to lower grain size as evidenced from FESEM study [1]. The optical band gap (*E*_g_) of α-Fe_2_O_3_ was calculated by plotting (*αhν*)^2^ or (*αhν*)^0.5^ as a function of photon energy (*hν*) using the Tauc relation [5]. Fig. 8d and e reveals the band gap energies (*E*_g_) determined from Tauc plots of two-step and one-step-annealed hematite photoanodes. The *E*_g_ values for direct and indirect optical transitions are 2.13–2.14 eV and 2.11 eV, respectively, which are consistent with the reported values. Although α-Fe_2_O_3_ is reported to exhibit an indirect (phonon-assisted) band gap transition around 1.9–2.2 eV, a few recent studies also reported a direct optical transition, which they attributed to quantum size effects or to the use of an electrochemical deposition method [6].
